# Individual and occupational correlates of work ability of Polish employees with visual impairments

**DOI:** 10.13075/ijomeh.1896.02762

**Published:** 2026

**Authors:** Katarzyna W. Binder-Olibrowska, Arkadiusz M. Jasiński, Magdalena A. Wrzesińska

**Affiliations:** 1 Medical University of Lodz, Department of Psychosocial Rehabilitation, Łódź, Poland; 2 University of Opole, Institute of Psychology, Opole, Poland

**Keywords:** social support, work ability, visual impairment, *Work Ability Index*, employees with disability, person-environment fit

## Abstract

**Objectives:**

Although occupational activity is a key determinant of human well-being, labor market participation among people with visual impairments remains low. While barriers to employment for individuals with low vision or blindness have been explored, little is known about how they perceive their own work ability. This study therefore aimed to assess the level of work ability in this population in Poland and identify its correlates, using the person-environment fit model as a theoretical framework.

**Material and Methods:**

A cross-sectional online survey was conducted among 67 participants with visual impairments, of whom 41 were partially sighted and 60% were women. The questionnaire collected sociodemographic, health, disability, and job-related information, as well as data on social support from colleagues and supervisors (*Copenhagen Psychosocial Questionnaire*). A dependent variable was measured with the *Work Ability Index*. Descriptive statistics, correlation, and hierarchical multiple regression analyses were performed.

**Results:**

Participants reported a generally good level of work ability (M±SD 37.54±6.46). Correlation analysis identified 5 variables significantly associated with work ability. In the regression model, 4 remained significant predictors: satisfaction with household income (β = 0.34, p < 0.001), self-rated health (β = 0.27, p < 0.01), presence of additional disability (β = –0.22, p < 0.01), and shift work (β = –0.23, p < 0.05). The model explained 62% of the variance in work ability (R² = 0.62). Additional analyses indicated that the examined variables explained more variance in the subjective-resource component of WAI than in its objective health component.

**Conclusions:**

The findings highlight key personal and occupational factors shaping work ability among employees with visual impairments. Considering these factors in workplace interventions may help sustain employment and promote occupational well-being in this group.

## Highlights

- Polish employees with visual impairment generally report good work ability.
- Income satisfaction and good health predict higher work ability.
- Additional disabilities and shift work increase the risk of reduced work ability.
- Targeted support is needed to enhance work ability in low-vision workers.

## INTRODUCTION

Visual impairment is associated with lower employment rates and difficulties in job retention [1,2]. In Poland, approx. 30% of people with disabilities are occupationally active [3], with this figure being probably even lower among the poor-sighted and blind. Low levels of professional productivity have negative consequences both in terms of quality of life of the people living with disabilities [2] and the worldwide economy [4]. Among the reasons why people with visual impairments experience difficulties in finding and retaining a job are health and disability-related concerns, employer attitudes, mobility limitations, financial viability, and the perception that one is too old to be employed [5]. Individual factors and psychosocial working conditions are also associated with beliefs in their own ability to work [6].

In this study, the authors analyzed the level of self-assessed work ability and factors related to it among Polish employees with visual impairments using the person-environment (P-E) fit model as a theoretical framework.

### Theoretical background

Person-environment fit model posits that the compatibility between an individual's attributes and environmental characteristics more accurately predicts behavior and health outcomes than considering either factor separately [7]. Optimal performance, including work ability, occurs when personal (e.g., needs) and environmental (e.g., resources) attributes are compatible [8]. In the context of occupational health and work ability, it is important to distinguish between 2 main types of fit: supplementary and complementary [9]. It could be assumed that complementary fit, which occurs when the person and the environment complement each other's deficiencies, may be particularly important in explaining work ability in research on workers with blindness and low vision. One type of complementary fit is a person-job fit [8]. Complementary fit has 2 fundamental dimensions which serve as explanatory mechanisms for work ability in the research:

– demands-abilities (D-A) fit – this refers to the degree to which an individual's abilities, knowledge, and skills match environmental demands such as workload, task complexity, and the physical demands of the job [8]. According to the P-E fit model, adequate D-A fit is fundamental to work ability because the individual can cope with the tasks they are asked to perform;
– needs-resources (N-R) fit – this occurs when the work environment provides resources that meet the employee's needs, desires, or goals. A high N-R fit is positively associated with job satisfaction and negatively associated with burnout and intention to leave the organization [10].

### Study aims and hypotheses

The aim of the study was to assess the level and correlates of subjective work ability in low-vision and blind people who are occupationally active. It was carried out by answering 2 research questions:

1. How do blind and partially sighted employees assess their ability to work?
2. What is the relationship between selected individual and occupational factors and the subjective ability to work among employees with visual impairments?

Work ability is defined as the dynamic balance between an individual's resources and job demands [11,12]. This balance reflects an individual's human resources (e.g., health, competencies, and values) and work factors (e.g., demands, organization, and environment). Maintaining this balance is essential for optimal work ability and health. Among workers with visual impairments, a mismatch between resources and demands may be a crucial predictor of work ability. First, a mismatch between demands and abilities can result from physical and cognitive barriers. Increased environmental demands (e.g., shift work) combined with reduced abilities (e.g., difficulty multitasking) can lead to an excessive workload, which may be associated with lower work ability. Second, a needs-resources mismatch occurs when the work environment fails to meet basic psychological and instrumental needs (e.g., lack of social support). This mismatch influences attitudes, motivation, and ultimately, the subjective dimensions of work ability.

The explanatory variables in the proposed study relate directly to individual characteristics, such as self-rated health, additional impairments, and household income satisfaction. They also relate to environmental factors, such as social support from colleagues and supervisors, as well as shift work. Social support from supervisors and co-workers is an important environmental resource. According to the P-E fit model, these resources satisfy employees’ fundamental psychological needs and maintain a balance between needs and resources [12]. Previous research shows that social support from supervisors and colleagues initiates a motivational process at work that is positively associated with work ability. This process is characterized by the activation of positive emotions at work, job engagement, and job satisfaction [13]. For employees who face unique adaptive challenges arising from their impairment, social support may be crucial for maintaining work ability. The first hypotheses (H) are:

– H1. Co-workers’ support is positively related to work ability;
– H2. Supervisory support is positively related to work ability.

Shift work is a common long-term work demand [14]. It forces employees to alter their natural circadian rhythm, which has been linked to health issues such as insomnia, obesity, and an elevated risk of cardiovascular disease and cancer [15]. Shift work increases environmental burden. For employees with ocular deficiency, this increased burden requires more adaptive effort, which can negatively affect work ability. Thus, work ability is compromised not only by primary visual impairment but also by the body's inability to adapt and compensate in demanding environmental conditions. Conversely, health has been identified as an important resource that is positively associated with work ability [16,17]. Employees with better health can more effectively meet job demands, experience greater job satisfaction, and consequently have higher work ability [18]. Based on the literature review, the following hypotheses were formulated:

– H3. Shift work is negatively related to work ability;
– H4. High level of self-rated health is positively related to work ability.

Previous studies have confirmed that multimorbidity is negatively associated with work ability [19]. According to the P-E fit model, health limitations reduce physical and mental resources, directly affecting work ability [8]. The authors argue that multimorbidity strengthens misfit because it reduces an employee's physical and mental work capacity and increases susceptibility to existing job demands. Job and life satisfaction are important outcomes of achieving P-E fit. Previous studies have confirmed that a higher P-E fit correlates positively with higher job and life satisfaction and negatively with turnover intention [20]. Job satisfaction is strongly associated with achieving N-R fit, or the feeling that the work environment provides sufficient resources to meet an employee's psychological and material needs [12]. Based on the theory and previous research, it was concluded that for people with health limitations like visual impairments, work ability is strongly linked to additional impairments and how satisfied they are with their income. Therefore, the following hypotheses are proposed:

– H5. Additional impairment is negatively related to work ability;
– H6. Income satisfaction is positively related to work ability.

## MATERIAL AND METHODS

### Study design

This cross-sectional study was conducted using an online questionnaire between August 2024 and April 2025. Due to the lack of data on the size of the study population in Poland, it was not possible to estimate representative sample size. The sample size was calculated using the *a priori* sample size calculator [21]. For a model with 8 predictors, assuming an effect size of f = 0.3, a desired statistical power of 0.85, and a probability level of p < 0.05, the required minimum sample size was N = 64. Attempts were made to reach as many people as possible by posting announcements about the study on internet forums and discussion groups for people with visual impairments, and by disseminating information about the study in the community, disability associations, and sports clubs, via online forums and discussion groups. The questionnaire was prepared in Microsoft Forms in a way that was accessible to people with vision loss, and before the actual study began, a pilot study was conducted in a group of people with varied ocular difficulties to assess its accessibility. The study used questions from validated questionnaires and questions of the authors’ own design. The questionnaire included a task to test attention: “If you read this sentence carefully, enter the correct result of the 12–5 equation.” The average time taken to complete the questionnaire was approx. 25 min.

### Ethics

The study protocol was verified by the Bioethics Committee of the Medical University of Lodz (RNN/151/24/KE), Łódź, Poland. The study was conducted with regard to the Declaration of Helsinki [22]. Participants gave their voluntary consent, had the option to withdraw at any time, and were assured of anonymity.

### Participants

The study included people who declared themselves to be ≥18 years, having a disability certificate due to visual impairment, and being employed or self-employed at the time of the study and who provided informed consent. People who answered the attention check question incorrectly were excluded. The analyses were conducted based on responses from 67 people, with a few data missing.

### Measures

#### Work ability

The *Work Ability Index* (WAI) is used for employees’ self-assessment of their ability to perform job, in relation to their personal resources (such as health and skills) as well as the job requirements [23]. In the Polish adaptation [24], the index consists of questions covered in 7 parts:

1) general ability to work,
2) ability to work in relation to physical and mental demand of the current work,
3) number of medical conditions diagnosed by a respondent or by a doctor, however only conditions diagnosed by a doctor are taken into account,
4) functional incapacity to work due to illness,
5) number of full days not worked due to health reasons (illness, treatment or examinations) in the last 12 months,
6) prognosis of ability to work for 2 years,
7) mental resources regarding satisfaction with performing daily activities, being active and hope in the last time.

Higher scores on each scale denote better functioning in an examined area, and the sum of results reflects a general WAI score. It is assessed as poor if the person gets 7–27 pts in total, moderate if they get 28–36 pts, good if they get 37–43 pts, and excellent if their total score is 44–49 pts. The reliability of the WAI in the present study was Cronbach's α = 0.65 for the overall questionnaire.

#### Social support in the workplace

*Copenhagen Psychosocial Questionnaire* (COPSOQ II) draws on many theoretical models to cover a wide range of psychosocial working conditions and different levels of human functioning in the workplace and is therefore applicable in various areas of the labor market. It is acceptable to use individual/detailed indicators contained in the questionnaire, which allows for a focus on selected aspects of the work environment [25]. In this study, the authors chose subscales regarding support from colleagues and supervisors (each containing 3 items) measuring the frequency with which an employee receives help and support from others in relation to problems at work. In each case, respondents could select 1 of 5 answers ranging from never/almost never to always or indicate that the question did not apply to them. The results obtained may range 0–100 pts. Reliability measured with Cronbach's α in the authors’ study was 0.81 for the support from colleagues and 0.90 regarding superiors’ support.

#### Self-rated health

As a measure of self-perceived health, the single item from the *World Health Organization Quality of Life –* BREF (WHOQOL-BREF) [26] was used. It had the following wording “Thinking about the last four weeks, are you satisfied with your health?” and 5 possible answers ranging from “very dissatisfied” (1 pt) to “very satisfied” (5 pts).

#### Sociodemographic, occupational and disability-related factors

In the final part of the survey, participants indicated their age, gender, place of residence, and education level. Respondents were also asked how they feel about their current household income by checking 1 of the following statements: “I live comfortably on my current income” (4 pts), “I manage on my current income” (3 pts), “I find it difficult to live on my current income” (2 pts), “I find it very difficult to live on my current income” (1 pt), “I prefer not to answer” (0 pts).

With regard to participants’ professional situation, questions were asked about: average weekly working hours, working shifts (yes/no), remote work (yes, full-time; yes, part-time; no), employment in a protected/closed labor market (e.g., protected workplace, vocational activity center) (yes, no, partially), and if a person is employed or self-employed. Respondents were also asked to determine whether their work is mainly mental or physical.

Considering disability, respondents were asked to specify whether they were poor-sighted or blind, indicate the level of disability (mild, moderate, severe) which best corresponded to their visual impairment, and whether they also had a certificate for a disability other than visual impairment (yes/no).

### Data analysis

The authors analyzed the data using IBM SPSS Statistics v. 26. After cleaning the dataset, observations that did not meet the inclusion criteria were excluded. Next, the descriptive statistics were calculated and the relationships between the sociodemographic and main study variables were examined. To test the hypotheses, a hierarchical regression analysis was conducted. The explanatory variables were education level, degree of visual impairment, social support from colleagues and supervisors, shift work, self-rated health, additional impairments, and household income satisfaction. The dependent variable was WAI. As an additional analysis, differences in WAI between selected groups were examined using Welch's t-tests due to unequal group sizes, and effect sizes were estimated using Cohen's d with 95% confidence intervals. Additionally, following suggestions from previous research indicating that WAI may consist of distinct components [27,28], the authors conducted an exploratory analysis separating it into 2 dimensions:

– an objective health component (scales 3–5 of the WAI),
– a subjective-resource component (scales 1, 2,6, and 7).

Hierarchical regression analyses were performed separately for these components using the same set of explanatory variables as in the main model.

## RESULTS

### Study group characteristics

The study group consisted mainly of individuals with low vision (61.2%) and women (60%), aged 18–75 years (mean [M] = 41.78, standard deviation [SD] = 10.95). Most participants had higher education (64.2%) and lived in cities with over 100 000 inhabitants (67.3%). Over 80% were employed in the open labor market, predominantly in the intellectual occupations (71.6%), and only 1 person was self-employed. Participants worked M±SD 34.57±9.05 h/week; nearly half worked remotely, and one-fifth on a shift basis. The perceived support from colleagues was M±SD 55.47±23.99 and from supervisors 51.45±30.94. Self-rated health averaged M±SD 3.33±0.91. Most respondents reported a severe degree of disability (73%), with additional disabilities present in 16.4%. The satisfaction with household income was M±SD 2.89±0.87.

#### Work ability

The WAI scores ranged 20–49, with an average overall score of M±SD 37.54±6.46, indicating that participants’ work ability was at a “good” level. Descriptive statistics for WAI dimensions are shown in Table 1.

**Table 1.** Values of *Work Ability Index* (WAI) and its dimensions in the workers with visual impairments, Poland, April 2025

WAI	Participants (N = 67) [n (%)]	WAI score (M±SD) [pts]
Level		
poor	5 (7.5)	
moderate	22 (32.8)	
good	26 (38.8)	
excellent	14 (20.9)	
Dimension		
1. Current work ability compared with the lifetime best (0–10 pts)		7.61±1.93
2. Work ability in relation to demands of the job (2–10 pts)		8.24±1.51
3. Number ofcurrent diseases diagnosed by a physician (0–5 pts)		4.29±2.20
≥5 diseases	13 (19.4)	
4 diseases	2 (3)	
3 diseases	9 (13.4)	
2 diseases	11 (16.4)	
1 disease	12 (17.9)	
no diseases	20 (29.9)	
4. Estimated work impairment due to diseases (1–7 pts)		4.43±1.32
“There is no hindrance”/“I have no diseases”	18 (26.9)	
“I am able to do my job, but it causes some symptoms”	15 (22.4)	
“I must sometimes slow down my work pace or change my work methods”	21 (31.3)	
“I must often slow down my work pace or change my work methods”	5 (7.5)	
“Because of my disease, I feel I am able to do only part-time work”	7 (10.4)	
“In my opinion, I am entirely unable to work”	1 (1.5)	
5. Sick leave during the past year (1–5 pts)		4.16±1.17
noneatall	37 (55.2)	
at the most 9 days	15 (22.4)	
10–24 days	8 (11.9)	
25–99 days	3 (4.5)	
100–365 days	4 (6.0)	
6. Personal prognosis of work ability 2 years from now (1–7 pts)		5.83±1.87
unlikely	5 (7.5)	
not certain	16 (23.9)	
relatively certain	46 (68.7)	
7. Mental resources, referring to life in general, both at work and during leisure time (1–4 pts)		2.92±0.87

The following independent variables were positively correlated with the overall WAI level: income satisfaction (r = 0.57), self-rated health (r = 0.57), supervisory support (r = 0.45, p < 0.001), co-workers support (r = 0.31, p < 0.01), degree of impairment (r = –0.27), and education level (r = 0.26, p < 0.05). In contrast, having an additional disability (r = –0.4, p < 0.001) and working shifts (r = –0.30, p < 0.05) were negatively associated with WAI. There were no significant correlations between WAI and age, gender, type of labor market, work setting, weekly working hours, or remote work (Table 2).

**Table 2.** Descriptive statistics and Pearson's correlations among the study variables, Poland, April 2025

Variable	M	SD	Sk	K	Pearson's r correlation													
					1	2	3	4	5	6	7	8	9	10	11	12	13	14
1. Age	41.78	10.95	0.68	0.34	—	0.20												
2. Gender	—	—	—	—	—	—												
3. Education level	—	—	—	—	0.01	−0.11	—											
4. Sheltered labor market	—	—	—	—	−0.07	−0.04	−0.23	—										
5. Work setting	—	—	—	—	0.13	0.11	0.45^***^	−0.13	—									
6. Degree of impairment	—	—	—	—	−0.02	0.12	−0.13	0.02	−0.20	—								
7. Co-workers support	55.47	23.99	−0.58	−0.07	−0.11	−0.16	−0.06	0.23	−0.16	0.12	—							
8. Supervisory support	51.45	30.94	−0.15	−1.12	0.08	0.11	0.09	0.19	0.04	0.11	0.58^***^	—						
9. Working time	34.57	9.05	−0.04	2.18	−0.20	0.22	0.05	−0.18	0.04	−0.19	−0.23	−0.15	—					
10. Remote work	—	—	—	—	−0.20	0.13	0.13	−0.30^*^	0.44^***^	0.00	−0.03	0.20	0.07	—				
11. Shift work	—	—	—	—	0.02	−0.10	−0.30^*^	−0.01	−0.44^***^	−0.16	0.12	−0.10	−0.02	−0.17	—			
12. Self-rated health	3.33	0.91	−0.46	−0.65	−0.09	0.04	0.23	0.02	0.16	0.38^**^	0.16	0.30^*^	0.14	0.16	−0.35^**^	—		
13. Additional impairment	—	—	—	—	0.13	0.05	−0.15	−0.09	0.01	−0.02	−0.27^*^	−0.32^*^	0.08	0.04	−0.01	−0.21	—	
14. Income satisfaction	2.89	0.87	−0.66	−0.001	0.06	0.22	0.19	−0.19	0.13	0.08	0.16	0.45^***^	0.10	0.25	−0.12	0.40^**^	−0.28^*^	—
15. Workability	37.54	6.46	−0.58	−0.10	−0.08	0.13	0.26^*^	−0.07	0.17	0.27^*^	0.31^**^	0.45^***^	0.09	0.22	−0.30^*^	0.57^***^	−0.42^***^	0.57^***^

K – kurtosis; Sk – skewness

Gender: 1 – women, 2 – men; education level: 1 – vocational, 2 – high school, 3 – graduate; sheltered labour market: 1 – no, 2 – partially, 3 – full sheltered workplace; work setting: 1 – physical/manual work, 2 – office/white collar work; degree of impairment: 1 – low, 2 – moderate, 3 – high; remote work: 1 – no, 2 – partially, 3 – full time; shift work: 1 – no, 2 – yes; additional impairment: 1 – no, 2 – yes.

* p < 0.05.

** p < 0.01.

*** p < 0.001.

— not calculated or not applicable.

To detect the predictive value of the independent variables in determining WAI of a study group, a hierarchical multiple regression was performed (Table 3). Variables were entered into the model in 4 steps. In subsequent models, a significant increase in explained variance was observed. Finally, model 4 explained 63% of the variance in the dependent variable (R² = 0.63). Household income satisfaction (β = 0.32, p < 0.01) and self-rated health (β = 0.23, p < 0.05) emerged as significant positive predictors of the overall WAI score, whereas having additional disability and working shifts were negative predictors (β = –0.21 and β = –0.20, respectively, p < 0.05).

**Table 3.** Hierarchical regression results for work ability, Poland, April 2025

Variable	B	SE(B)	95% Cl for B	β	R^2^	ΔR^2^	F(df)
Model 1					0.19	0.19^**^	6.99(2,57)^**^
constant	16.09	5.70	4.66–27.52				
education level	3.91	1.47	0.96–6.85	0.31^**^			
degree of impairment	4.06	1.43	1.19–6.94	0.33^**^			
Model 2					0.43	0.23^***^	8.13(5,54)^***^
constant	20.18	6.52	7.10–33.26				
education level	2.95	1.34	0.27–5.64	0.23^*^			
degree of impairment	2.76	1.30	0.13–5.38	0.22^*^			
co-workers support	0.06	0.03	−0.008–0.12	0.23			
supervisory support	0.05	0.02	0.002–0.11	0.27^*^			
shift work	−3.58	1.84	−7.28–0.10	−0.22			
Model 3					0.50	0.07^**^	8.94(6,53)^***^
constant	18.62	6.17	6.25–31				
education level	2.07	1.30	0.16–1.59	0.16			
degree of impairment	1.19	1.35	−0.53–4.68	0.09			
co-workers support	0.05	0.03	−0.011–0.11	0.20			
supervisory support	0.04	0.02	−0.007–0.09	0.21			
shift work	−2.43	1.78	−6.01–1.13	−0.15			
self-rated health	2.34	0.83	0.66–4.02	0.33^**^			
Model 4					0.63	0.12^**^	10.87(8,51)^***^
constant	24.26	6.24	11.74–36.79				
education level	1.32	1.16	−1–3.65	0.10			
degree of impairment	0.78	1.19	−1.61–3.18	0.06			
co-workers support	0.05	0.02	−0.001–0.11	0.22			
supervisory support	0.005	0.02	−0.04–0.05	0.02			
shift work	−3.32	1.58	−6.49–(–0.14)	−0.20^*^			
self-rated health	1.60	0.75	0.08–3.13	0.23^*^			
additional impairment	−3.57	1.53	−6.66–(–0.49)	−0.21^*^			
income satisfaction	2.44	0.76	0.90–3.98	0.32^**^			

* p < 0.05.

** p < 0.01.

*** p < 0.001.

### Additional analyses

Additional analyses confirmed that participants not working shifts and those without additional impairment had significantly higher WAI scores than their counterparts (Table 4).

**Table 4.** Results of Welch's t-test comparing *Work Ability Index* by shift work status and additional impairment, Poland, April 2025

Variable	Participants [n]	WAI score [pts]			t	df	d	95% Cl
		M	SD	Me				
Shift work								
no	54	38.48	5.94	39.25	2.20^*^	15.99	0.72	0.185–9.46
yes	13	33.65	7.34	35.00				
Additional impairment								
no	56	38.74	5.75	40	3.35^**^	13.01	1.16	2.59–11.97
yes	11	31.45	6.74	32				

** p < 0.01;

* p < 0.05.

Additional analyses also examined whether the predictors of work ability differed between components of the WAI (Table 5). In 2 separate regression models, the subjective-resource component showed a substantially higher explained variance (R² = 0.60) than the objective health component (R² = 0.29). Income satisfaction, self-rated health, co-workers’ support, and additional impairment were significant predictors of the subjective-resource component, whereas only supervisory support was associated with the objective health component. The 2 components were moderately correlated (r = 0.32, p = 0.008).

**Table 5.** Regression results for *Work Ability Index* (WAI) – objective health component and subjective-resource component, Poland, April 2025

Variable	B	SE	95% Cl for B	β	R^2^	F(df)
Objective health component (dimensions 3,4,5 of WAI)						
constant	10.24	3.82	2.57–17.91		0.29	2.65(8,51)^*^
education level	0.00	0.71	−1.42–1.43	0.00		
degree of impairment	0.88	0.73	−0.59–2.35	0.16		
co-workers support	0.00	0.02	−0.03–0.04	0.02		
supervisory support	0.03	0.02	0.00–0.06	0.34^*^		
shift work	−1.26	0.97	−3.20–0.69	−0.18		
self-rated health	0.02	0.46	−0.91–0.95	0.01		
additional impairment	−0.75	0.94	−2.64–1.14	−0.10		
income satisfaction	0.25	0.47	−0.69–1.19	0.08		
Subjective-resource component (dimensions 1,2,6, and 7 of WAI)						
constant	14.03	5.05	3.90–24.16		0.60	9.59(8,51)^***^
education level	1.32	0.94	−0.56–3.20	0.14		
degree of impairment	−0.09	0.97	−2.04–1.85	−0.01		
co-workers support	0.06	0.02	0.01–0.10	0.27*		
supervisory support	−0.03	0.02	−0.07–0.01	−0.16		
shift work	−2.06	1.28	−4.63–0.50	−0.17		
self-rated health	1.59	0.61	0.35–2.82	0.29^*^		
additional impairment	−2.83	1.24	−5.32–(–0.34)	−0.22^*^		
income satisfaction	2.19	0.62	0.94–3.43	0.38^***^		

* p < 0.05;

*** p < 0.001.

## DISCUSSION

In the study, the authors evaluated the level of WAI in the self-assessment of Polish employees with visual impairments and examined its personal and occupational correlates.

The theoretical framework of this project is based on the assumption that self-assessment of work ability can serve as a subjective indicator of P-E fit. By combining assessments of health, competence and work context, it reflects the perceived fit between an employee's resources and the requirements of the job [8]. Several surveys show that this perception is strongly predictive of work performance, sickness absence, return to work, retirement, and well-being [29,30]. This is consistent with a P-E fit model, which posits that perceived misfit leads to tension and reduced performance.

The average level of WAI demonstrated by the study's respondents in relation to the norms [24] can be described as good. It is very similar to that reported previously in the general Polish working population [31] and higher than those obtained by respondents with low vision surveyed by Belevska et al [6]. From the perspective of P-E fit model, a good WAI level in the study's group, similar to that of the general Polish population, may result from the employees’ personal resources (health, functional abilities, skills, motivation) being reasonably well matched to the demands of the environment (job tasks, pace of work, physical/mental demands), which indicates that a good P-E fit has been achieved. However, according to the authors of the WAI [24], achieving a good WAI level still indicates a need for support in order to improve work ability. To this end, it is worth learning about the correlates of work ability that may be relevant when planning interventions.

In the initial hypotheses, the authors assumed that support received at work would have positive correlations with work ability, as it has already been reported in employees with low vision [6]. While the previous study assessed overall support from both colleagues and superiors as a single factor, the authors’ study analyzed these sources separately. The current findings show that only support from superiors significantly predicts WAI (confirmed H2, but not H1). A crucial role of a supervisor for work-related outcomes has already been reported [32,33]. The supervisor plays a key role in organizing the workplace, caring for the health and wellbeing of employees, and creating an organizational culture that promotes the use of employee potential and cooperation. The feeling of having the support of a supervisor makes it easier for employees to report difficulties and needs, which, especially for people with disabilities, can be crucial to adapting to working conditions and creating an inclusive environment. Translating this into P-E fit model, supervisors control and shape the basic characteristics of the environment that determine fit. Support from supervisors reduces the mismatch between job demands and employee capabilities by reducing stress and enabling adjustments that restore fit for employees with disability-related limitations and maintain or improve their ability to work. On the other hand, support from colleagues can contribute to the psychosocial climate, strengthening the sense of belonging, counteracting isolation, or reducing the risk of burnout [34]. However, compared to support from a supervisor, it has less impact on changing formal job requirements or access to organizational resources. Given the limited understanding of how different types of workplace support affect the work ability of individuals with ocular limitations, further research is needed. Future investigations should explore the distinct mechanisms through which various sources of support, particularly supervisory versus peer support, impact work ability, and how these relationships may vary across different types of health impairments and occupational settings.

Hypothesis 3, in which the authors assumed a negative relationship between working shifts and work ability, was confirmed. Although, to the authors’ best knowledge, there is a lack of research on the impact of shift work on employees with visual impairments, it can be assumed that the consequences described for other populations also apply to them. Numerous publications indicate that shift work leads, inter alia, to circadian rhythm disruption and cognitive disorders, like impaired concentration, psychomotor vigilance, and memory [35]. People with visual impairments rely on other senses, attention, and memory to perform tasks, navigate, and access information that their sighted colleagues do with their eyesight. This heightens the demands on working memory and attention (higher cognitive load), which may further slow performance and increase fatigue when faced with prolonged job demands or multitasking [36]. These higher cognitive demands and the risk of circadian rhythm disturbances observed in people with visual impairments [37] may be additive or multiplicative factors that reduce their work ability while working shifts. The observed negative relationship between shift work and work ability among employees with visual impairments highlights a critical area for further investigation. Future research should aim to identify the mechanisms through which shift work affects this population and explore targeted interventions, such as tailored support systems or adaptive workplace policies, that can enhance coping strategies and promote sustainable work ability

Two further hypotheses were also confirmed – H4 concerned the positive relationship between self-rated health, and H5 the negative correlation between having >1 disability and work ability.

A higher number of chronic diseases in different populations is associated with a lower work ability [19,38], and higher risk of a transition to partial or full retirement [39]. In a study examining the ability to remain in employment after losing one's sight while already employed, better self-assessed health proved to be a facilitating factor, while having more chronic diseases was associated with higher risk of ceasing the professional activity [40]. Systematic review by Daniels et al. [41] indicated that additional disabilities negatively affected the labor market participation of people with visual impairments.

Multiple disabilities may intensify barriers at work related to access, speed, or task flexibility, resulting in greater accommodation needs. On the other hand, higher self-assessment of health may be related to fewer functional limits, more energy, and better capacity to meet job demands. Also notable is that the impact of multi-morbidity on work ability may depend on the employee's capacity to cope with job demands [38] and on the type of disease. As research shows, mental disorders co-occurring with physical ones exacerbate the negative impact on work ability [19,38]. Additionally, in some studies, health status was not related to employment among people who are visually impaired [42], and additional disability correlated positively with their job satisfaction [33], which may indicate that employees with such difficulties can still effectively use their potential in a professional context. These mixed findings underscore the need for further research to explore how different combinations of disabilities interact to influence work ability, what protective factors – such as resilience, workplace culture, or coping strategies – support positive outcomes, and how tailored interventions and workplace support can mitigate challenges. Longitudinal studies are especially needed to track how work ability evolves over time in people with multiple health conditions and to identify effective strategies for sustaining their employment and well-being.

To sum up, in the light of P-E model, resources such as energy, functional capacity, and cognitive alertness are reduced by shift work and multiple disabilities, and conversely, they increase with a higher self-rated health status. This results in a respective reduction or increase in the mismatch between job demands and personal capabilities.

In line with the assumptions of H6, household income satisfaction proved to be positively related to work ability. Although research on the relationship between income and work capacity is inconclusive [17], the authors assumed the link would be significant for workers with visual impairments, who often face greater economic burdens and lower earnings than non-disabled workers [1,43]. Generally, financial satisfaction corresponds with life satisfaction, lower stress levels, and a better perception of control – mechanisms that protect functioning at work [44]. Among employees with disabilities, financial security increases their perceived ability to cope with job demands by promoting access to assistive devices, healthcare, or transport [45], reducing chronic stress, maintaining mental and physical cognitive abilities, increasing perceived fairness, and facilitating motivation and job satisfaction. All of these resources promote P-E fit, reduce the gap between demands and resources, and improve perceptions of one's ability to work.

An important finding of the present study is that some participants reported financial difficulties despite being occupationally active, suggesting that employment does not necessarily guarantee economic security among people with visual impairment. This pattern may reflect the phenomenon of in-work poverty among people with disabilities, which has been linked to lower wages, labor-market inequalities, and disability-related extra costs [46]. In the Polish context, supporting financial well-being may therefore involve not only improving income stability and job quality but also reducing disability-related financial burdens through workplace accommodations, access to assistive technologies, or employment support instruments, such as workplace adjustment programs financed by the State Fund for Rehabilitation of Persons with Disabilities (Państwowy Fundusz Rehabilitacji Osób Niepełnosprawnych – PFRON) [47]. Such measures may enhance employees’ resources, improve P-E fit, and help sustain work ability over time.

Additional analyses suggested that the variables examined in this study explained substantially more variance in the subjective-resource component of the WAI than in its objective health component. The moderate correlation between these components indicates that they are related but may reflect partly distinct aspects of work ability. This may be particularly relevant in the case of employees with visual impairments, for whom subjective resources and workplace conditions may partly compensate for health-related limitations. However, the present study was not designed to validate the factorial structure of the WAI, and the sample size does not allow reliable factor-analytic verification. Further research should examine the multidimensional structure of the WAI and its relationships with both health indicators and psychosocial workplace factors.

### Strengths and limitations

Several limitations of the study should be acknowledged. Due to its cross-sectional design, the study does not allow for causal inferences, limiting the authors’ ability to determine the directionality of observed relationships. The relatively small sample size restricts the generalizability of the findings and prevents subgroup analyses, such as comparisons between the blind and partially sighted participants or between different combinations of co-occurring conditions. Additionally, the reliance on self-reported data introduces the possibility of response bias. However, the study also has notable strengths. This study is the first in Poland and one of the few worldwide to explore the work ability of employees with visual impairments offering novel insights into a neglected area of occupational health research. The use of an online survey tailored to the needs of respondents with visual impairments made it possible to reach a population group that is usually underrepresented and difficult to access, thereby increasing the inclusiveness of the research. The statistical model applied was appropriate for the available data and allowed for the identification of significant predictors of WAI. These findings lay the groundwork for future longitudinal and intervention studies aimed at understanding the complex interplay between different types of disabilities and employment outcomes, and at developing tailored support strategies for individuals with multi-morbidity in the workplace.

## CONCLUSIONS

Polish low-vision and blind workers demonstrate a good level of work ability and require measures to maintain or improve it. The analysis identified several factors that are significantly related to work ability in the study group and that have been analyzed with the P-E fit model.

Higher self-rated health and greater satisfaction with household income appear to serve as protective factors for work ability, likely by enhancing personal resources and reducing the gap between job demands and individual capacity. In contrast, the presence of multiple disabilities and engagement in shift work emerge as significant risk factors, potentially diminishing ability to work by impairing productivity, cognitive functioning, and recovery processes. These conditions may contribute to a persistent mismatch between the worker's capabilities and occupational requirements. Furthermore, supervisor support plays a critical role in sustaining work ability. Given that supervisors influence task allocation, scheduling, and the provision of formal accommodations, their support can directly enhance the alignment between an employee's abilities and job demands, thereby promoting better work outcomes.

Understanding the factors associated with subjective work ability among workers with visual impairments is essential for designing effective preventive strategies aimed at sustaining their work ability and long-term employability. The authors’ findings highlight the need to introduce workplace strategies that foster P-E fit and minimize the mismatch between personal factors and job demands. Such strategies should encompass health promotion initiatives, support for financial well-being, individualized adaptations of working conditions, and strong supervisory support. Such measures are in line with national and international legal frameworks that require employers to create inclusive workplaces with appropriate facilities for employees with disabilities [48].
